# Immune checkpoint inhibitors associated inflammatory disease of central nervous system: Case report and systemic review

**DOI:** 10.1097/MD.0000000000043552

**Published:** 2025-08-01

**Authors:** Ting-Ting Yang, Ze-Yi Wang, Pen-Ju Liu, Guang-Zhi Liu

**Affiliations:** a Department of Neurology, Beijing Anzhen Hospital, Capital Medical University, Beijing, China.

**Keywords:** case report, central nervous system demyelinating disease, immune checkpoint inhibitors, neurological immune-related adverse effects

## Abstract

**Rationale:**

The use of immune checkpoint inhibitors (ICIs) has greatly improved the outcomes of cancer. However, ICI-induced immune-related adverse events have been reported, among which neurological immune-related adverse events are rare but potentially life-threatening. To analyze the characteristics of ICI-induced inflammatory disease of the central nervous system (CNS), we reported the first case induced by tislelizumab and conducted a systematic review of 33 cases.

**Patient concerns:**

A 65-year-old male patient who received 4 cycles of tislelizumab for lung squamous carcinoma developed myelitis, demyelinating encephalopathy, and peripheral neuropathy overlap syndrome. Case reports and case series that described ICI-associated inflammatory disease of the CNS in the PubMed and Embase databases were searched up to October 2022.

**Diagnoses:**

The patient was diagnosed with ICI-associated neuroimmune overlap syndrome, including myelitis, peripheral neuropathy, and multifocal demyelinating encephalopathy.

**Interventions:**

The patient was treated with intravenous methylprednisolone.

**Outcomes:**

The symptoms alleviated gradually. Twenty-six publications that described 33 cases with ICI-associated inflammatory disease of the CNS were reviewed. In addition to the present case, only 8 cases with both brain and spinal cord lesions induced by ICIs were reported. Intravenous steroids were the first-line therapy, while plasmapheresis, intravenous immunoglobulin, cyclophosphamide, and some monoclonal antibodies may be effective for steroid-refractory neurological immune-related adverse events. Thirteen patients experienced relapse, and 4 patients died, one of whom committed suicide.

**Lessons:**

ICI-associated inflammatory disease of the CNS is very rare, but we should be aware of and detect it to treat timely manner and achieve a good prognosis.

## 1. Introduction

Immune checkpoint inhibitors (ICIs) have been considered as an emerging standard-of-care for a variety of cancers, greatly improve the prognosis of advanced cancer.^[1]^ ICIs mainly target the programmed death protein-1 (PD-1), programmed death-ligand 1 (PD-L1), or cytotoxic-T lymphocyte-antigen-4 (CTLA-4) to regulate the immune response.^[2]^ Due to their effect of immune system, especially T cell activity, immune-related adverse effects (irAEs) have always been reported. Forty percent patients treated with ICIs developed irAEs, such as dermatitis, pneumonia, colitis, and hepatitis.^[3]^ Neurological irAEs (nirAEs) are rare,^[4]^ including myositis, Guillain-Barré syndrome, myasthenic syndromes, encephalitis, cranial neuropathies, meningitis, central nervous system (CNS) demyelinating diseases, myelitis, etc.^[5]^

Tislelizumab, an anti-PD-1 antibody, was approved in 2019^[6]^ to treat hematological cancers and metastatic solid tumors, including Hodgkin lymphoma, urothelial carcinoma, gastric and esophageal cancer, etc.^[6]^ The irAEs caused by tislelizumab were generally mild to moderate,^[7]^ including pneumonitis,^[8]^ enteritis,^[9]^ and multiple-organs injuries.^[10]^ NirAEs of tislelizumab have not been reported.

We presented a rare case with lung squamous cell carcinoma who developed tislelizumab associated peripheral neuropathy, myelitis, demyelinating encephalopathy. Besides, we conducted a systematic review of published cases that described ICIs associated inflammatory disease of CNS.

## 2. Materials and methods

The clinical data of this patient was collected from the electronic medical record system at the time of hospital admission and was kept confidential and informed consent was waived due to the retrospective observational case report.

### 2.1. Search strategy

A comprehensive literature review was conducted to gather case reports or case series that reported patients with ICIs associated CNS inflammatory lesions. Electronic databases consisting of PubMed (Table S1, Supplemental Digital Content, https://links.lww.com/MD/P522) and Embase (Table S2, Supplemental Digital Content, https://links.lww.com/MD/P523) were searched using MeSH terms including “ICIs,” “ipilimumab,” “nivolumab,” “pembrolizumab,” “atezolizumab,” “durvalumab,” “avelumab,” “tislelizumab,” “myelitis,” “myelitis, transverse,” “neuromyelitis optica,” “encephalomyelitis,” “spinal cord diseases,” and “leukoencephalopathies.” Articles published up to October 2022 were included.

### 2.2. Eligibility criteria

All the included articles reported cases with myelitis, encephalitis, or encephalomyelitis related to ICIs. The eligible article types are case reports, case series, conference abstracts, and letter to editor. Publications that were not written in English were excluded. Neurological complications without a definite causal relationship with ICIs were excluded. Cases without significant pathological changes in the spinal cord or brain were excluded. If a publication included multiple neurological diseases associated with ICIs, we only included cases presenting myelitis, transverse myelitis, encephalomyelitis, meningoencephalomyelitis, neuromyelitis optica, myeloradiculitis, leukoencephalopathy, and demyelinating disease of the CNS.

### 2.3. Study selection and data extraction

**Figure 1.**
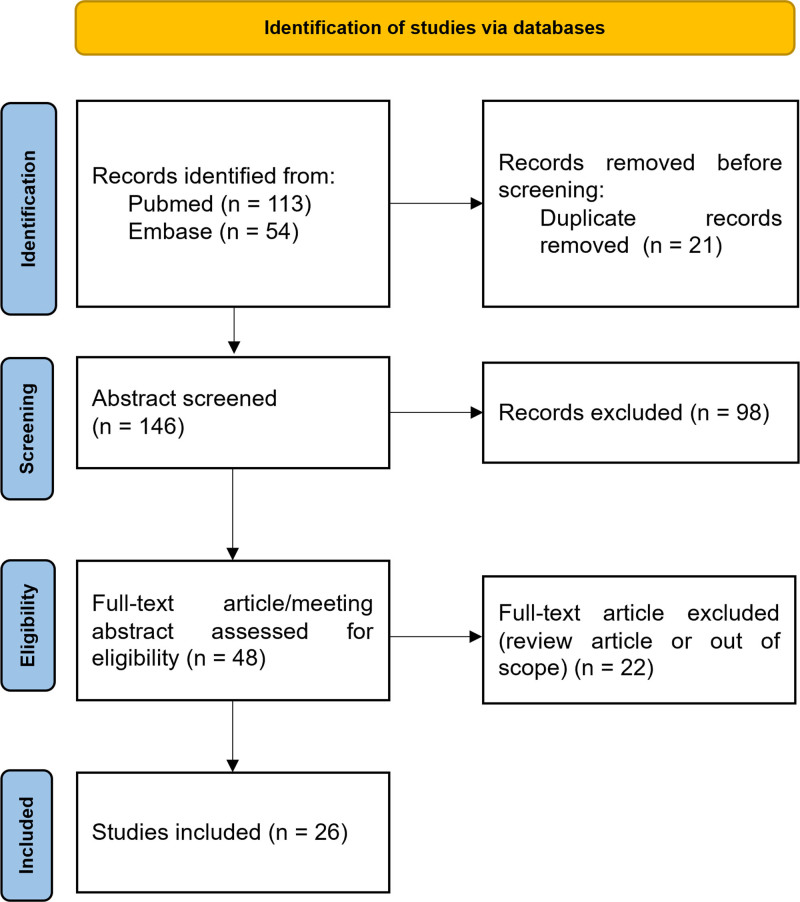
Flowchart of the inclusion process. From Page et al.^[11]^ For more information, visit: http://www.prisma-statement.org/.

Duplicated articles were excluded by identifying titles, authors, and years of publication (Fig. 1). Articles with irrelevant contents or undefined diagnosis were excluded by reviewing the full text or abstract. Two reviewers participated in the inclusion process of eligible articles independently. Any disagreement of the eligibility of a case was settled with consensus by another reviewer. Clinical characteristics, including demographics, ICIs cycles and clinical presentations, radiological changes, cerebrospinal fluid (CSF) examinations, treatments, and outcomes of the neurological complications, were analyzed.

## 3. Results

### 3.1. Case report

A 65-year-old male patient, was admitted to our hospital with complaint of “foot numbness for 8 months, right eyelid ptosis, and general numbness and weakness for more than 3 months, with aggravation for 20 days.” He was diagnosed as squamous carcinoma in the right lung 8 months ago, and received 4 cycles of tislelizumab plus paclitaxel and nedaplatin (per month) and surgical resection at our hospital (Fig. S1, Supplemental Digital Content, https://links.lww.com/MD/P524). The patient has a 20-years smoking history, without any familial history, or history of infections or exposure to toxic substances and heavy metals.

The numbness initially began at bilateral toes after the first cycle of treatment, which lasted for several hours. The numbness reoccurred at both feet following the second cycle and self-relieved after about 1 day. Ten days following the fourth cycle of tislelizumab, the patient gradually presented with right ptosis, slurred speech, dysphagia, dizziness and walking instability. The patient also showed progressive numbness and weakness, predominantly in the left side. Generalized myasthenia gravis was suspected at the local hospital. He was given intravenous immunoglobulin (IVIg) (0.4 g/kg/day for 5 days) and intravenous methylprednisolone (80 mg/day for 5 days, 60 mg/day for 6 days), followed by oral prednisone (40 mg/day). The ptosis and numbness were relieved, but slurred speech, dysphagia, and generalized weakness persisted. Twenty days before admission to our hospital, the patient developed pneumonia, accompanied by worsened generalized numbness and weakness. He couldn’t walk without aid. Neurological examination revealed sensory loss at bilateral face, left limbs and T2 level. Muscle atrophy was found at the proximal limbs. The muscle strength of bilateral upper limb was grade 5−, left lower limb grade 4−. Expanded disability status scale was 6.5.

**Figure 2.**
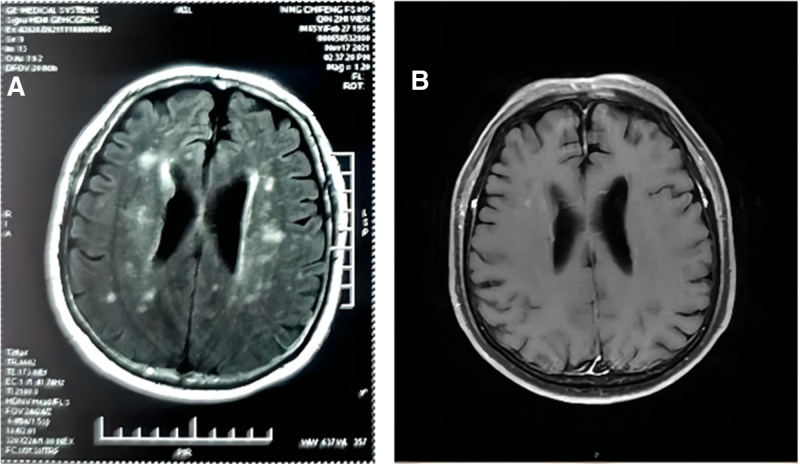
Cranial MRI showed multifocal demyelinating changes of the white matter in the periventricular region. (A) Cranial MRI. (B) Contrast cranial MRI. MRI = magnetic resonance imaging.

**Figure 3.**
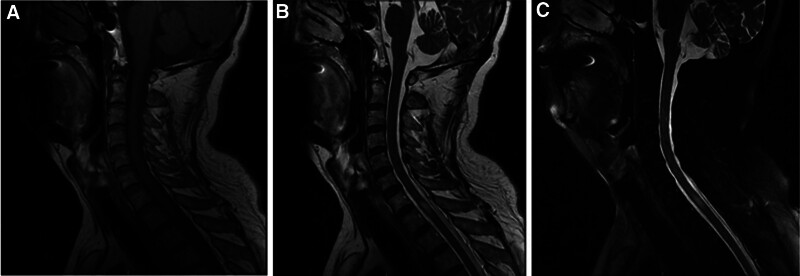
MRI indicated lesions of the spinal cord at T1 level. (A) T1WI. (B) T2WI. (C) T2 fat-saturation sequence. MRI = magnetic resonance imaging.

Cranial magnetic resonance imaging (MRI) showed multifocal periventricular demyelinating lesions (Fig. 2A), with small patchy enhancement (Fig. 2B). Spinal MRI presented patchy T_2_ and T_2_ fat-saturation hyperintense at T1 level (Fig. 3). Neostigmine test was negative. Electromyography showed diffuse motor and sensory conduction impairment, as well as axon injury. The autoimmune screen and paraneoplastic antibodies were normal. Lumbar punctures showed elevated pressure (200 mmH2O), protein level (104.12 mg/dL), glucose level (5.08 mmol/L), and normal white blood cells number (3/mm^3^) (Table 1). Positron emission tomography/ computed tomography (PET/CT) showed no lung cancer recurrence or metastasis. He was diagnosed as ICIs associated neuroimmune overlap syndrome including myelitis, peripheral neuropathy and multifocal demyelinating encephalopathy.

**Table 1:** Cerebrospinal fluid examination of the patient.

Cerebrospinal fluid	Result	Reference
Glucose	5.08 mmol/L	2.5–4.4
Protein	104.12 mg/dL	15–45
ADA	1.6 U/L	4–20
Cl	120.1 mmol/L	120–130
Cells	3/mm^3^	
WBC	3/mm^3^	
Mononuclear cells	15.80%	
Multinuclear cells	84.20%	
Cytological examination	Tumor cells not found	
OCB	Negative	
24 h intrathecal IgG synthesis rate	3.16 mg/24 h	<7
IgG index	0.54	≤0.7
Anti-GFAP antibody	Negative	Negative
Anti-AQP4 antibody	Negative	Negative
Paraneoplastic antibodies	Negative	Negative
Peripheral neuropathy antibodies	Negative	Negative
Autoimmune encephalitis antibodies	Negative	Negative

ADA = adenosine deaminase, AQP4 = aquaporin 4, GFAP = glial fibrillary acidic protein, IgG = Immunoglobulin G, OCB = oligoclonal bands, WBC = white blood cells.

The patient was given intravenous methylprednisolone (240 mg daily for 3 days, tapering by half every 3 days), followed by oral prednisone 40 mg daily. The symptoms of numbness and weakness were improved gradually. On the 10th day post methylprednisolone start, the physical examination showed that the sensory level was reduced to T10 level, muscle strength of both sides was restored to level 5. After discharge, the patient continued to take prednisone and the dosage was gradually reduced and the symptoms alleviated gradually. Repeated MRI at the local hospital revealed reduced lesions both in the brain and spinal cord.

### 3.2. Systematic review of the literature

Twenty six publications^[12–37]^ with 33 cases of ICIs associated leukoencephalopathy and/or myelitis were identified. The clinical characteristics of the 33 included cases are listed in Table 2.

**Table 2:** Cases identified in the published cases (including 29 articles and 36 cases).

Source	Age/sex	Cancer	ICIs (cycles)	Radiation	Symptoms	Radiology	CSF WBC (cells/μL), Pro (mg/dL)	Diagnosis	Tx	Relapse	Outcomes
Liao et al, 2014^[28]^	62/M	MM	Ipilimumab (3)	No	Weakness, paresthesia, sphincter dysfunction	T9–10 myelitis	Glu: ↑ (137 mg/dL), WBC: ↑ (28), Pro: N, OCB: (−)	TM	High-dose steroids	No	Improved, melanoma progressed
O’Kane et al, 2014^[32]^	58/M	MM	Ipilimumab (2)	Yes (T6–8)	weakness, sphincter dysfunction	T7-L1 →C4/5-cauda equina	Glu: N, WBC: ↑ (16), Pro: ↑ (50–57.6), OCB: (−)	Myelitis	steroids pulsed weekly; IVIG	Yes	progressed
Zabad et al, 2015^[37]^	46/M	MM	Ipilimumab (3)	Yes (brain)	Vision blurring, numbness	entire spinal cord	WBC: ↑ (201), Pro: ↑ (2-fold), OCB: (+)	Opticospinal inflammatory disease	High-dose steroids	Yes	Improved
Abdallah et al, 2016^[12]^	45/F	MM	Ipilimumab (2)	Yes (brain)	Paraplegia, sphincter dysfunction, sensory loss	C5, T3/4, T6/7, T11/12, conus, cauda equine, sacral roots, CE (+)	Glu: N, WBC: ↑ (303), Pro: ↑ (310), OCB: (−)	Necrotic myelopathy	Oral steroids; infliximab	Yes	Not improved
Chang et al, 2018^[17]^	68/M	MM	Ipilimumab + nivolumab (4) →pembrolizumab (1)	Yes (T7–10, brain)	paresthesia, ataxia, sphincter dysfunction	T5–10, CE (+) →T3–11→T6–10	Pro: ↑ (99), OCB: type IV	TM	IV MP; PE; CTX; Infliximab	No	Improved, but died
Wilson et al, 2018^[36]^	35/M	HL	Pembrolizumab (2)	No	Tetraparesis, paresthesia, sphincter dysfunction	Pons to lower thoracic spine	Glu: N, WBC: ↑ (24), Pro: N	LETM	IV MP, PE, IVIG	No	Improved
Makkawi et al,2019^[19]^	53/F	MM	Nivolumab (6 m)	No	sphincter dysfunction, neuropathic pain, weakness	T1–12→C6-conus	WBC: ↑, Pro: ↑, OCB: (+)	LETM	IV MP, PE	Yes	Improved
–	54/F	MM	Nivolumab + ipilimumab (14 w)	No	weakness, paresthesia, sphincter dysfunction	C2-conus	WBC: ↑, Pro: ↑, OCB: (+)	LETM	IV MP, PE	Yes	Improved
Poretto, 2019^[34]^	73/M	CcRcc	nivolumab	No	Weakness, sphincter dysfunction	T3-conus, CE (+)	WBC: N, Pro: ↑ (80)	Myelitis; possible NMOSD	High-dose steroids	No	Mildly improved
Brahmbhatt and Dixit, 2020^[16]^	61/F	MM	Nivolumab + Ipilimumab	No	Weakness, paresthesia, ataxia	Cervicomedullary junction-conus	Glu: N, WBC: ↑ (75), Pro: ↑ (125)	LETM	IV MP	Yes	Improved
Nowosielski, 2020^[31]^	47/M	MM	Nivolumab (13), ipilimumab (3)	No	Facial palsy, paraparesis, sphincter dysfunction, blurred vision	Entire spinal cord, periventricular, CE (+)	WBC: ↑ (120), Pro: ↑, OCB: (+)	Encephalomyelitis; optic neuritis	IV MP, Rituximab	Yes	Complete recovery
Charabi et al, 2021^[23]^	63/M	NSCLC	Pembrolizumab (2)	No	sphincter dysfunction, painful dysphagia, paraparesis	Cervical, thoracic, CE (+) → T5–11	Glu: ↑, WBC: ↑ (63), Pro: ↑ (80), OCB: (−)	LETM	Oral steroids→ IV MP, PE	Yes	Died
Bolz et al, 2021^[15]^	55/M	MM	Ipilimumab (2)	No	paresthesia, paresis, clonus	C1, C3–5, CE (+)	WBC: ↑ (8), Pro: ↑ (72)	TM	IV MP	No	Improved
Picca et al, 2021^[20]^	57/M	NSCLC	Nivolumab (12)	Yes (thoracic)	Paraparesis, neuropathic pain, sphincter dysfunction	C7-T4, T11–12, CE (+)	WBC: ↑ (88), Pro: ↑ (376), OCB: (+)	Myeloradiculitis	Oral prednisone →IV MP + PE + CTX	Yes	Slightly improved
	62/F	NSCLC	Nivolumab (7)	Yes (T4)	Paraparesis, paresthesia, sphincter dysfunction	Entire spinal cord, CE (+)	NA	Myelitis	IV MP	No	Died
	16/F	Mesenteric IMT	Pembrolizumab (19)	No	Paraparesis, ataxia, paresthesia, radicular pain, sphincter dysfunction	C4–5, C7-T3, T9–12, CE (+)	WBC: N, Pro: N	Myeloradiculitis	IV MP	No	Recovery
	59/M	NSCLC	Pembrolizumab (5)	No	Tetraparesis, sphincter dysfunction, neck stiffness, neuropathic pain, dysphagia, altered consciousness	C1-T10, bulbar, Periventricular, leptomeningeal, CE (+)	WBC: ↑ (900), Pro: ↑ (520)	Meningoencephalomyelitis	IV MP, PE, Natalizumab	No	Improved
	61/F	NSCLC	Pembrolizumab (5)	No	Paraparesis, ataxia, paresthesia	C3–4, CE (-)	WBC: ↑ (105), Pro: ↑ (75), OCB: (+)	myelitis	IV MP, CTX	No	Not improved
	57/M	NSCLC	Nivolumab (51)	Yes	Paraparesis, ataxia, paresthesia, radicular pain, bladder hyperactivity	C3–6, T2–3, T8–11, periventricular, thalamocapsular, frontoinsular cortex, CE (+)	WBC: N, Pro: ↑ (109), OCB: (+)	Encephalomyelitis; demyelinating polyradiculoneuritis	IV MP, IVIg, PE	Yes	Not improved
	58/M	Melanoma	Nivolumab and Ipilimumab (4)	No	Paraplegia, paresthesia, sphincter dysfunction	C2, C3, C7-T2, T4–7, T8-conus, brain hemispheric, cerebellar, CE (+)	WBC: ↑ (115), Pro: ↑ (257), OCB: (−)	Meningoencephalomyeloradiculitis	IV MP, PE, Tocilizumab	No	Slightly improved
Moodie et al, 2022^[29]^	68/M	NSCLC	Durvalumab (1 yr)	Yes (lung)	Pain, paraparesis, sphincter dysfunction, paresthesia	C4-T11, CE (+) → T3–7 →C4-T4	WBC: ↑ (8), Pro: ↑ (48), OCB: (-)	LETM	High-dose steroids, PE, CTX, methotrexate	Yes	Died
Shimada et al, 2020^[21]^	63/F	NSCLC	Pembrolizumab (1)	Yes (brain)	paraparesis, paresthesia, sphincter dysfunction	C4-T1, CE (+)	WBC: ↑ (9), Pro: ↑ (48), OCB: (−)	NMOSD	IV MP, PE	No	Improved
Kunchok et al, 2019^[24]^	63/F	SCLC	Atezolizumab (3)	No	Paraparesis, paresthesia, Sphincter dysfunction	C5-midthoracic, CE (+)	Glu: N, WBC: ↑ (46), Pro: ↑ (105)	Paraneoplastic myelopathy	IV MP, CTX	No	Improved
Nasralla and Abboud, 2020^[30]^	30/F	HL	Nivolumab (3)	No	Paraparesis, Sphincter dysfunction, tonic spasms, Vision loss	Cervical, thoracic, → entire cord, optic chiasm, optic nerve, CE (+)	1st: N 2nd: WBC: ↑ (345), Pro: ↑ (516), OCB: (−)	myelitis	IV MP, PE, Rituximab	Yes	Improved
Narumi et al, 2018^[22]^	75/M	NSCLC	Nivolumab (1)	No	Paraparesis, paresthesia, Sphincter dysfunction	C5–6; T12-L1	Glu: ↓(40 mg/dL), WBC: ↑ (1195), Pro: ↑ (380.9)	NMOSD	High-dose steroids, PE	No	Improved
Yuen et al, 2020^[26]^	58/F	Thymic squamous cell carcinoma	Pembrolizumab (24)	No	No	Numerous punctate enhancing foci	Glu: N, WBC: N, Pro: N, OCB: (+)	CLIPPERS like	No	No	Complete resolution
Owen and Fung, 2022^[33]^	67/F	NSCLC	Pembrolizumab (4)	Yes (T3/4–T10/11)	Weakness, Sphincter dysfunction	T4/5 → T4/5-T10/T11, CE (+)	NA	myelitis	IV MP, IVIg, PE, MMF	Yes	Slightly improvement
Durães et al, 2018^[18]^	58/F	MM	Pembrolizumab (3)	No	Paresthesia, weakness	Periventricular, cervico-dorsal, CE (+)	Pro: ↑, OCB: (+)	Drug-induced inflammatory disease of the CNS	IV MP, PE	No	Almost recovery
Bjursten et al, 2021^[14]^	67/M	MM	Nivolumab + ipilimumab (4)	No	Paraparesis, unconsciousness, Sphincter dysfunction	Brain, brainstem, cerebellum, spinal cord	Glu: N, WBC: ↑ (16), OCB: (+)	Acute disseminated encephalomyelitis	IV MP, CTX, MMF, IVIg	No	Improved
Feng and O’Byrne, 2016^[27]^	66/M	NSCLC	Pembrolizumab (2)	Yes (brain)	Dysphasia, cognitive decline	Cerebral hemisphere	N	Encephalopathy	High-dose steroids	No	Improved
Strik et al, 2017^[35]^	53/M	NHL	Nivolumab (NA)	No	Diplopia, ataxia, dysarthria	Lateral ventricle, midbrain, brain stem	Glu: N, WBC: ↑ (16), OCB: (+)	CNS inflammation	IV MP, IVIg, CTX	No	Suicide
Pillonel et al, 2019^[25]^	44/M	MM	Ipilimumab (1) → Nivolumab (11)	Yes (brain)	Asymptomatic	Multiple white matter, open ring CE (+)	Glu: N, WBC: ↑ (14), Pro: ↑ (59.4), OCB: (−)	Demyelinating disease	No	No	Improved
Basin et al, 2021^[13]^	59/M	NSCLC	Pembrolizumab (5)	No	Altered consciousness, weakness, hypothermia, bulbar palsy, Sphincter dysfunction	Bulb, cervical spine, CE (+)	Glu: N, WBC: ↑ (900), Pro: ↑ (520)	Meningoencephalomyelitis	IV MP; PE; natalizumab	No	Improved

CcRcc = clear cell renal cell carcinomas, CE = contrast enhancement, CTX = cyclophosphamide, F = female, Glu = glucose, HL = Hodgkin lymphoma, IV MP = intravenous methylprednisolone, M = male, m = month, MIMT = mesenteric inflammatory myofibroblastic tumor, MM = metastatic melanoma, MMF = mycophenolate mofetil, N = normal, NHL = non-Hodgkin lymphoma, NMO = neuromyelitis optica, NSCLC = non-small-cell lung cancer, OCB = oligoclonal bands, PE = plasmapheresis, Pro = protein, SCLC = small-cell lung cancer, TM = transverse myelitis, w = week, WBC = white blood cells.

### 3.3. Demographic

Age of the included cases ranged from 16 to 75 years (median = 58, IQR = 10), and 39.4% (13/33) were female patients. The primary cancer included melanoma or metastatic melanoma (14, 42.4%), non-small-cell lung cancer (12, 36.4%), Hodgkin lymphoma (2, 6.1%). Other cancers such as small-cell lung cancer, clear cell renal carcinoma, mesenteric inflammatory myofibroblastic tumor and thymic squamous cell carcinoma, were less common, each of which was 1 (3%). The included ICIs regimen included pembrolizumab (11, 33.3%), nivolumab (8, 24.2%), ipilimumab (5, 15.2%), atezolizumab (1, 3.0%), durvalumab (1, 3.0%). There were 5 patients (15.2%) used nivolumab and ipilimumab combination, one of whom changed to pembrolizumab. Two patients who used ipilimumab or nivolumab monotherapy and change to another one. The reported ICIs cycles ranged from 1 to 51 (medium = 4, IQR = 5). Twelve (36.4%) patients received radiation therapy previously. There was also 1 patient who had well controlled sicca syndrome.

### 3.4. Clinical presentation and examination

There were only 2 patients who didn’t have any symptoms. The most common symptoms were varying degrees of paralysis (27, 81.8%), paresthesia (18, 54.5%) and sphincter dysfunction (24, 72.7%). Pain (6, 18.2%), including neuropathic pain or radicular pain, and ataxia (6, 18.2%) were also reported. Other symptoms that had been reported were abnormal vision (3, 9.1%), altered consciousness (3, 9.1%), dysphagia (2, 6.1%), etc. For radiography, 21 patients (63.6%) had lesions in spinal cord, while 8 patients (24.2%) had both spinal cord and brain lesions including leukoencephalopathy and encephalitis. There were also 4 patients (12.1%) with lesions limited in the brain. Among the patients with spinal cord lesions, the most common is transverse myelitis. There was also 1 patient with optic chiasm and optic nerve involved. Among 22 patients who reported contrast MRI, 21 had enhancement (95.5%). CSF analysis showed inflammatory alterations in most of the cases, including elevated protein levels (24/29, 82.8%) and pleocytosis (24/29, 82.8%). Most patients had mild (≤100 cells/μL) pleocytosis (13/27, 48.1%), and only 1 patient had more than 1000 cells/μL. For patients with elevated protein, there were equal patients (10/25, 40%) for protein concentration higher than 100 mg/dL or lower than 100 mg/dL. Most of the patients didn’t have known antibodies. Only 1 patient reported with rheumatoid factor and CCP antibody. One patient had glial fibrillary acidic protein antibodies, while 2 patients had AQP4 antibodies. One patient had been screened to have 1 unknown antibodies.

### 3.5. Treatment and outcome

Two asymptomatic patients recovered spontaneously without receiving therapies. All other 31 patients received steroids, 3 of which started with oral steroids and 2 escalated to intravenous steroids. Among the 27 patients receiving intravenous high-dose steroid treatment, 18 (66.7%) cases showed a significant improvement or almost full recovery of neurologic function, and 3 (11.1%) cases were slightly improved, while 2 (7.41%) cases progressed or did not improve, and 4 (14.8%) cases died or suicided. However, of the 4 patients receiving oral steroids or weekly pulsed steroids, only 1 (25%) patient demonstrated mild improvement, whereas 2 (50%) patients progressed, and 1 (25%) patient died. These above results suggest that the dosage variations could potentially influence treatment outcome. Nevertheless, more evidence is needed to determine if higher or lower doses of steroid correlate with any differential effects. Other first line therapies included plasmapheresis (15/33, 45.5%), intravenous immunoglobulin (6/33, 18.2%), cyclophosphamide (7/33, 21.2%). Biological agents or immunosuppressor agents (2 cases with natalizumab, 2 cases with rituximab, 2 cases with mycophenolate mofetil, 2 cases with infliximab, 1 case with tocilizumab, and 1 case with methotrexate) were also used in some cases. Twenty four (72.7%) patients were clinically improved with varying degrees. Three (9.1%) patients did not improve, and 1 patient progressed. Five (25.2%) patients died and one of whom suicided. Relapses were observed in 13 (39.4%) patients. Among the 13 relapse patients, 2 patients did not improve and 2 patients died, and 1 progressed.

## 4. Discussion

It is estimated that the incidence of nirAEs is no more than 3% of all irAEs.^[38]^ The high-grade nirAEs were rarer, with the incidence <1%,^[4]^ but needed prompt recognition due to the severity. CNS is less commonly affected compared with peripheral nervous system or neuromuscular junction.^[39]^ The CNS nirAEs include myelitis, encephalitis, meningitis, cranial neuropathies, and CNS demyelinating diseases.^[5]^ In this article, we reported a rare case with myelitis, demyelinating encephalopathy and peripheral neuropathy induced by ICIs. To our knowledge, it is also the first reported nirAEs case caused by tislelizumab. To analyze the characteristics of cases with CNS inflammatory disease induced by ICIs, we conducted a systemic review.

The mechanisms of action about ICIs are interrupting the inhibitory signals of the cytotoxic-T lymphocytes to restore or increase their antitumor activities.^[40]^ An abnormal T cell response to antigens that are presented from normal cells is believed to be the cause of ICIs associated irAEs.^[41]^ Since both CTLA-4 and PD-1 are widely expressed on T lymphocytes, irAEs secondary to ICIs have been extensively described.^[42]^ Although the mechanisms of nirAEs are not fully understood and no evidence that ICIs directly affect the nervous system has been reported till now, oligodendrocytes and microglial cells that express PD-L1^[43]^ and endocrine cells that express CTLA-4^[44,45]^ are possible targets of ICIs.

Differential diagnosis of nirAEs following ICIs is complex. In our case, we have done paraneoplastic antibodies and PET/CT to excluded tumor progression. Other typical inflammatory disease of nervous system, such as multiple sclerosis, neuromyelitis optica spectrum disorders (NMOSD), myasthenia gravis has been excluded without typical clinical course or antibodies. However, some cases had been reported with known antibodies,^[19–22]^ unknown antibodies^[23]^ or paraneoplastic antibody.^[24]^ Vascular and infectious diseases could also be ruled out according to the clinical presentation.

Similar to our case, there are 8 patients reported to have both brain and spinal cord lesions. But the most common is pure myelitis, consistent with the most common clinical presentation with paralysis, paresthesia, and sphincter dysfunction. There are only 2 asymptomatic patients who had lesions limited in the brain,^[25,26]^ which may indicate that brain lesions may be easily overlooked. Enhancement characterized by punctate or patchy is more common in radiology examination. CSF examination is featured by mild to moderate elevation of the protein level and white blood cells, suggesting the CNS inflammation induced by immune responses.

Except for 2 patients who recovered spontaneously, all other patients received steroids, mainly intravenous methylprednisolone. Plasmapheresis and intravenous immunoglobulin were also widely used therapies, which may accelerate the clearance of ICIs and pathogenic antibodies.^[46]^ 8 patients received steroids alone, among which 7 patients showed improvement, and 1 died. For refractory or relapse patients, some immunosuppressants such as mycophenolate mofetil (MMF), Cyclophosphamide (CTX) and biological agents (natalizumab, rituximab, infliximab and tocilizumab) maybe the alternative choice, but we should be alert to the risk of primary cancer progression caused by immunosuppressants. More than one-third (13, 39.4%) patients experienced relapses, and they were less likely to improve and easily to progressed. We should be aware of nirAEs of CNS for patients who received ICIs and treat them timely to acquire better prognosis.

## 5. Conclusion

NirAEs of CNS are rare, especially brain and spinal cord affected meanwhile. Here we reported a case presenting with myelitis, demyelinating encephalopathy and peripheral neuropathy induced by tislelizumab, who responded well to steroid. Due to the possible poor prognosis, we should be aware of nirAEs, and treat them timely. Steroid was used widely. Meanwhile, plasmapheresis, immunoglobulin and biological agents were also good choices especially for steroid refractory cases.

## Acknowledgments

We thank the patient for the contributions and agreement to publish clinical data in journals.

## Author contributions

**Conceptualization:** Ting-Ting Yang, Pen-Ju Liu, Guang-Zhi Liu.

**Data curation:** Ting-Ting Yang, Ze-Yi Wang.

**Formal analysis:** Ting-Ting Yang, Ze-Yi Wang.

**Investigation:** Ting-Ting Yang, Ze-Yi Wang.

**Methodology:** Ting-Ting Yang, Ze-Yi Wang.

**Project administration:** Guang-Zhi Liu.

**Resources:** Pen-Ju Liu, Guang-Zhi Liu.

**Software:** Ting-Ting Yang, Ze-Yi Wang.

**Supervision:** Pen-Ju Liu, Guang-Zhi Liu.

**Validation:** Pen-Ju Liu, Guang-Zhi Liu.

**Visualization:** Ting-Ting Yang, Guang-Zhi Liu.

**Writing – original draft:** Ting-Ting Yang.

**Writing – review & editing:** Guang-Zhi Liu.

## Supplementary Material


