# Supplementary material for: Immune checkpoint inhibitors associated inflammatory disease of central nervous system: Case report and systemic review
Source: Medicine (Baltimore). 2025 Aug 1;104(31):e43552. doi: 10.1097/MD.0000000000043552 (PMC12324009; doi:10.1097/MD.0000000000043552)
Supplement: Supplementary file 1 [file medi-104-e43552-s001.docx]

**Supplementary Table 1.** The detailed search strategies used on PubMed database.

| Databases | Search level | Search terms | Filter | Results |
| --- | --- | --- | --- | --- |
| PubMed | 1 | (immune checkpoint inhibitors) AND (associated inflammatory disease) | 2010-2022 | 521 |
| PubMed | 2 | (immune checkpoint inhibitors) AND (associated inflammatory disease) | Year: 1996 – 2020,  Free full text | 327 |
| PubMed | 3 | (immune checkpoint inhibitors) AND (associated inflammatory disease) | Clinical Trial | 35 |
| PubMed | 4 | (immune checkpoint inhibitors) AND (associated inflammatory disease) | Free full text, Clinical Trial | 20 |
| PubMed | 5 | (central nervous system) AND (immune checkpoint inhibitors)) AND (associated inflammatory disease) | 2003-2022 | 11 |
| PubMed | 6 | immune checkpoint inhibitors | 1991-2022 | 35089 |
| PubMed | 7 | (ipilimumab) AND (immune checkpoint inhibitors) | 2005-2022 | 3963 |
| PubMed | 8 | (ipilimumab) AND (immune checkpoint inhibitors) AND （case report) | 2007-2022 | 863 |
| PubMed | 9 | (ipilimumab) AND (immune checkpoint inhibitors) AND （case series) | 2012-2022 | 93 |
| PubMed | 10 | (ipilimumab) AND (immune checkpoint inhibitors) AND (conference abstracts) | 2012-2022 | 11 |
| PubMed | 11 | (ipilimumab) AND (immune checkpoint inhibitors) AND (letter to editor) | 2019-2022 | 2 |
| PubMed | 12 | (nivolumab) | 2006-2022 | 8968 |
| PubMed | 13 | (nivolumab)AND (immune checkpoint inhibitors) | 2010-2022 | 6891 |
| PubMed | 14 | (nivolumab)AND (immune checkpoint inhibitors) AND (case report) | 2013-2022 | 1938 |
| PubMed | 15 | (nivolumab)AND (immune checkpoint inhibitors) AND (case series) | 2015-2022 | 145 |
| PubMed | 16 | (nivolumab)AND (immune checkpoint inhibitors) AND (conference abstracts) | 2016-2022 | 15 |
| PubMed | 17 | (nivolumab)AND (immune checkpoint inhibitors) AND (letter to editor) | 2016-2022 | 9 |
| PubMed | 18 | (pembrolizumab) | 2006-2022 | 8217 |
| PubMed | 19 | (pembrolizumab) AND (immune checkpoint inhibitors) AND (case report) | 2013-2022 | 1495 |
| PubMed | 20 | (pembrolizumab) AND (immune checkpoint inhibitors) AND (case series) | 2016-2022 | 132 |
| PubMed | 21 | (pembrolizumab) AND (immune checkpoint inhibitors) AND (conference abstracts) | 2018-2022 | 10 |
| PubMed | 22 | (pembrolizumab) AND (immune checkpoint inhibitors) AND (letter to editor) | 2019-2022 | 8 |
| PubMed | 23 | (atezolizumab) | 2006-2022 | 2680 |
| PubMed | 24 | (atezolizumab) AND (immune checkpoint inhibitors) | 2012-2022 | 1977 |
| PubMed | 25 | (atezolizumab) AND (immune checkpoint inhibitors) AND (case report) | 2016-2022 | 249 |
| PubMed | 26 | (atezolizumab) AND (immune checkpoint inhibitors) AND (case series) | 2017-2022 | 25 |
| PubMed | 27 | (atezolizumab) AND (immune checkpoint inhibitors) AND (conference abstracts) | 2018-2022 | 5 |
| PubMed | 28 | (atezolizumab) AND (immune checkpoint inhibitors) AND (letter to editor) | 2018-2022 | 11 |
| PubMed | 29 | (durvalumab) | 2006-2022 | 1330 |
| PubMed | 30 | (durvalumab) AND (immune checkpoint inhibitors) | 2012-2022 | 654 |
| PubMed | 31 | (durvalumab) AND (immune checkpoint inhibitors) AND (case report) | 2016-2022 | 73 |
| PubMed | 32 | (durvalumab) AND (immune checkpoint inhibitors) AND (case series) | 2016-2022 | 15 |
| PubMed | 33 | (durvalumab) AND (immune checkpoint inhibitors) AND (conference abstracts) | 2018-2022 | 5 |
| PubMed | 34 | (durvalumab) AND (immune checkpoint inhibitors) AND (letter to editor) | Up to 2022 | 0 |
| PubMed | 35 | avelumab | 2006-2022 | 858 |
| PubMed | 36 | (avelumab)AND (immune checkpoint inhibitors) | 2015-2022 | 460 |
| PubMed | 37 | (avelumab)AND (immune checkpoint inhibitors) AND (case report) | 2016-2022 | 28 |
| PubMed | 38 | (avelumab)AND (immune checkpoint inhibitors) AND (case series) | 2019-2022 | 9 |
| PubMed | 39 | (avelumab)AND (immune checkpoint inhibitors) AND (letter to editor) | Up to 2022 | 0 |
| PubMed | 40 | tislelizumab | 2006-2022 | 147 |
| PubMed | 41 | (tislelizumab) AND (immune checkpoint inhibitors) | 2018-2022 | 52 |
| PubMed | 42 | (tislelizumab) AND (immune checkpoint inhibitors) AND (case report) | 2020-2022 | 24 |
| PubMed | 43 | (tislelizumab) AND (immune checkpoint inhibitors) AND (case series) | 2021-2022 | 4 |
| PubMed | 44 | (tislelizumab) AND (immune checkpoint inhibitors) AND (conference abstracts) | Up to 2022 | 0 |
| PubMed | 45 | (tislelizumab) AND (immune checkpoint inhibitors) AND (letter to editor) | Up to 2022 | 0 |
| PubMed | 46 | myelitis | 1865-2022 | 30057 |
| PubMed | 47 | (myelitis) AND (immune checkpoint inhibitors) | 2014-2022 | 37 |
| PubMed | 48 | (myelitis) AND (immune checkpoint inhibitors) AND (case report) | 2014-2022 | 16 |
| PubMed | 49 | (myelitis) AND (immune checkpoint inhibitors) AND (case series) | 2021-2022 | 1 |
| PubMed | 50 | (myelitis) AND (immune checkpoint inhibitors) AND (conference abstract) | Up to 2022 | 0 |
| PubMed | 51 | (myelitis) AND (immune checkpoint inhibitors) AND (letter to editor) | Up to 2022 | 0 |
| PubMed | 52 | myelitis, transverse | 1931-2022 | 6762 |
| PubMed | 53 | (myelitis, transverse) AND (immune checkpoint inhibitors) | 2014-2022 | 20 |
| PubMed | 54 | (myelitis, transverse) AND (immune checkpoint inhibitors) AND (case report) | 2014-2022 | 11 |
| PubMed | 55 | (myelitis, transverse) AND (immune checkpoint inhibitors) AND (case series) | 2021-2022 | 1 |
| PubMed | 56 | (myelitis, transverse) AND (immune checkpoint inhibitors) AND (conference abstract) | Up to 2022 | 0 |
| PubMed | 57 | (myelitis, transverse) AND (immune checkpoint inhibitors) AND (letter to editor) | Up to 2022 | 0 |
| PubMed | 58 | neuromyelitis optica | 1937-2022 | 5994 |
| PubMed | 59 | (neuromyelitis optica) AND (immune checkpoint inhibitors) | 2018-2022 | 12 |
| PubMed | 60 | (neuromyelitis optica) AND (immune checkpoint inhibitors) AND (case report) | 2018-2022 | 6 |
| PubMed | 61 | (neuromyelitis optica) AND (immune checkpoint inhibitors) AND (case series) | Up to 2022 | 0 |
| PubMed | 62 | (neuromyelitis optica) AND (immune checkpoint inhibitors) AND (conference abstract) | Up to 2022 | 0 |
| PubMed | 63 | (neuromyelitis optica) AND (immune checkpoint inhibitors) AND (Letter to editor) | Up to 2022 | 0 |
| PubMed | 64 | encephalomyelitis | 1915-2022 | 33243 |
| PubMed | 65 | (encephalomyelitis) AND (immune checkpoint inhibitors) | 1995-2022 | 51 |
| PubMed | 66 | (encephalomyelitis) AND (immune checkpoint inhibitors) AND (case report) | 2013-2022 | 4 |
| PubMed | 67 | (encephalomyelitis) AND (immune checkpoint inhibitors) AND (case series) | Up to 2022 | 0 |
| PubMed | 68 | (encephalomyelitis) AND (immune checkpoint inhibitors) AND (Letter to editor) | Up to 2022 | 0 |
| PubMed | 69 | (encephalomyelitis) AND (immune checkpoint inhibitors) AND (conference abstract) | Up to 2022 | 0 |
| PubMed | 70 | spinal cord diseases | 1828-2022 | 163866 |
| PubMed | 71 | (spinal cord diseases) AND (immune checkpoint inhibitors) | 1998-2022 | 25 |
| PubMed | 72 | (spinal cord diseases) AND (immune checkpoint inhibitors) AND (case report) | 2011-2022 | 18 |
| PubMed | 73 | (spinal cord diseases) AND (immune checkpoint inhibitors) AND (case series) | Up to 2022 | 0 |
| PubMed | 74 | (spinal cord diseases) AND (immune checkpoint inhibitors) AND (conference abstract) | Up to 2022 | 0 |
| PubMed | 75 | (spinal cord diseases) AND (immune checkpoint inhibitors) AND (Letter to editor) | Up to 2022 | 0 |
| PubMed | 76 | eukoencephalopathies | 1945-2022 | 40325 |
| PubMed | 77 | (eukoencephalopathies) AND (immune checkpoint inhibitors) | 1995-2022 | 106 |
| PubMed | 78 | (eukoencephalopathies) AND (immune checkpoint inhibitors) AND (case report) | 2016-2022 | 29 |
| PubMed | 79 | (eukoencephalopathies) AND (immune checkpoint inhibitors) AND (case series) | 2020-2022 | 5 |
| PubMed | 80 | (eukoencephalopathies) AND (immune checkpoint inhibitors) AND (conference abstract) | Up to 2022 | 0 |
| PubMed | 81 | (eukoencephalopathies) AND (immune checkpoint inhibitors) AND (letter to editor) | Up to 2022 | 0 |
