# Supplementary material for: Immune checkpoint inhibitors associated inflammatory disease of central nervous system: Case report and systemic review
Source: Medicine (Baltimore). 2025 Aug 1;104(31):e43552. doi: 10.1097/MD.0000000000043552 (PMC12324009; doi:10.1097/MD.0000000000043552)
Supplement: Supplementary file 2 [file medi-104-e43552-s002.docx]

**Supplementary Table 2.** The detailed search strategies used on Embase database.

| History | Search | Results |
| --- | --- | --- |
| #52 | eukoencephalopathies AND immune AND checkpoint AND inhibitors AND ([conference abstract]/lim OR [letter]/lim) AND [embase]/lim AND [<1966-2022]/py | 0 |
| #51 | eukoencephalopathies AND immune AND checkpoint AND inhibitors | 0 |
| #50 | eukoencephalopathies AND immune AND checkpoint AND inhibitors | 0 |
| #49 | eukoencephalopathies | 0 |
| #48 | #47 AND ('case report'/de OR 'case study'/de) | 3 |
| #47 | #46 AND (2016:py OR 2018:py OR 2019:py OR 2020:py OR 2021:py OR 2022:py) AND ('case report'/de OR 'case study'/de) AND ('conference abstract'/it OR 'letter'/it) | 3 |
| #46 | spinal AND cord AND diseases AND immune AND checkpoint AND inhibitors | 14 |
| #45 | spinal AND cord AND diseases | 25,484 |
| #44 | #43 AND 'case report'/de | 1 |
| #43 | #42 AND (2010:py OR 2013:py OR 2015:py OR 2016:py OR 2017:py OR 2018:py OR 2019:py OR 2020:py OR 2021:py OR 2022:py) AND 'case report'/de AND 'conference abstract'/it | 1 |
| #42 | encephalomyelitis AND immune AND checkpoint AND inhibitors | 43 |
| #41 | encephalomyelitis | 42607 |
| #40 | #39 AND (2019:py OR 2020:py OR 2021:py OR 2022:py) AND 'case report'/de AND 'conference abstract'/it | 0 |
| #39 | #38 AND (**2019**:py OR **2020**:py OR **2021**:py OR **2022**:py) AND **'case report'**/de AND **'conference abstract'**/it | 14 |
| #38 | **（neuromyelitis** AND **optica）** | 12031 |
| #37 | #36 AND **'case report'**/de | 6 |
| #36 | #35 AND (**2016**:py OR **2018**:py OR **2019**:py OR **2020**:py OR **2021**:py OR **2022**:py) AND (**'case report'**/de OR **'case study'**/de) AND **'conference abstract'**/it | 6 |
| #35 | #34 AND (**2016**:py OR **2018**:py OR **2019**:py OR **2020**:py OR **2021**:py OR **2022**:py) AND (**'case report'**/de OR **'case study'**/de) AND **'conference abstract'**/it | 47 |
| #34 | **（myelitis,** AND **transverse）** | 6383 |
| #33 | #32 AND **'case report'**/de | 7 |
| #32 | #31 AND (**2015**:py OR **2016**:py OR **2017**:py OR **2018**:py OR **2019**:py OR **2020**:py OR **2021**:py OR **2022**:py) AND (**'case report'**/de OR **'case study'**/de) AND (**'conference abstract'**/it OR **'letter'**/it) | 7 |
| #31 | **myelitis** AND **immune** AND **checkpoint** AND **inhibitors** | 133 |
| #30 | **myelitis** | 16365 |
| #29 | #28 AND **'case report'**/de | 4 |
| #28 | #27 AND (**2017**:py OR **2018**:py OR **2019**:py OR **2020**:py OR **2021**:py OR **2022**:py) AND **'case report'**/de AND **'conference abstract'**/it | 4 |
| #27 | **（tislelizumab）** AND **（immune** AND **checkpoint** AND **inhibitors）** | 938 |
| #26 | **tislelizumab** | 2811 |
| #25 | #24 AND **'case report'**/de | 34 |
| #24 | #23 AND (**2014**:py OR **2015**:py OR **2016**:py OR **2017**:py OR **2018**:py OR **2019**:py OR **2020**:py OR **2021**:py OR **2022**:py) AND **'case report'**/de AND (**'conference abstract'**/it OR **'letter'**/it) | 34 |
| #23 | **avelumab** AND **immune** AND **checkpoint** AND **inhibitors** | 3378 |
| #22 | **avelumab** | 7296 |
| #21 | #20 AND **'case report'**/de | 66 |
| #20 | #19 AND (**2013**:py OR **2014**:py OR **2015**:py OR **2016**:py OR **2017**:py OR **2018**:py OR **2019**:py OR **2020**:py OR **2021**:py OR **2022**:py) AND **'case report'**/de AND (**'conference abstract'**/it OR **'letter'**/it) | 66 |
| #19 | **（durvalumab）** AND **（immune** AND **checkpoint** AND **inhibitors）** | 5224 |
| #18 | **durvalumab** | 13707 |
| #17 | #16 AND **'case report'**/de | 97 |
| #16 | #15 AND (**2013**:py OR **2014**:py OR **2015**:py OR **2016**:py OR **2017**:py OR **2018**:py OR **2019**:py OR **2020**:py OR **2021**:py OR **2022**:py) AND **'case report'**/de AND (**'conference abstract'**/it OR **'letter'**/it) | 97 |
| #15 | **（atezolizumab）** AND **（immune** AND **checkpoint** AND **inhibitors）** | 7556 |
| #14 | #13 AND **'case report'**/de | 386 |
| #13 | #12 AND (**2012**:py OR **2013**:py OR **2014**:py OR **2015**:py OR **2016**:py OR **2017**:py OR **2018**:py OR **2019**:py OR **2020**:py OR **2021**:py OR **2022**:py) AND **'case report'**/de AND (**'conference abstract'**/it OR **'letter'**/it) | 386 |
| #12 | **atezolizumab** | 20720 |
| #11 | #11 AND (**2014**:py OR **2015**:py OR **2016**:py OR **2017**:py OR **2018**:py OR **2019**:py OR **2020**:py OR **2021**:py OR **2022**:py) AND (**'case report'**/de OR **'case study'**/de) AND (**'conference abstract'**/it OR **'letter'**/it) | 2124 |
| #10 | #10 AND (**2014**:py OR **2015**:py OR **2016**:py OR **2017**:py OR **2018**:py OR **2019**:py OR **2020**:py OR **2021**:py OR **2022**:py) AND (**'case report'**/de OR **'case study'**/de) AND (**'conference abstract'**/it OR **'letter'**/it) | 10829 |
| #9 | **pembrolizumab** | 48047 |
| #8 | #7 AND (**2010**:py OR **2011**:py OR **2012**:py OR **2013**:py OR **2014**:py OR **2015**:py OR **2016**:py OR **2017**:py OR **2018**:py OR **2019**:py OR **2020**:py OR **2021**:py OR **2022**:py) AND (**'case report'**/de OR **'case study'**/de) AND (**'15th croatian oncology congress'**:nc OR **'17th world conference of the international association for the study of lung cancer, iaslc 2016'**:nc OR **'18th world conference on lung cancer of the international association for the study of lung cancer, iaslc 2017'**:nc OR **'2016 annual meeting of the american society of clinical oncology, asco 2016'**:nc OR **'2017 annual meeting of the american society of clinical oncology, asco'**:nc OR **'2018 annual meeting of the american association for cancer research, aacr 2018'**:nc OR **'2018 annual meeting of the american society of clinical oncology, asco 2018'**:nc OR **'2018 asco-sitc clinical immuno-oncology symposium'**:nc OR **'2019 annual meeting of the american society of clinical oncology, asco 2019'**:nc OR **'2020 annual meeting of the american society of clinical oncology, asco 2020'**:nc OR **'2020 annual scientific meeting of the american college of gastroenterology, acg 2020'**:nc OR **'2020 genitourinary cancers symposium'**:nc OR **'2020 world conference on lung cancer'**:nc OR **'2021 world conference on lung cancer worldwide virtual event'**:nc OR **'22th national congress of italian association of medical oncology, aiom'**:nc OR **'33rd annual meeting and pre-conference programs of the society for immunotherapy of cancer, sitc 2018'**:nc OR **'35th annual meeting and pre-conference programs of the society for immunotherapy of cancer, sitc 2020'**:nc OR **'36th annual meeting of the society for immunotherapy of cancer`s, sitc 2021'**:nc OR **'37th annual meeting of the society for immunotherapy of cancer`s, sitc 2022'**:nc OR **'42nd esmo congress (esmo 2017)'**:nc OR **'43rd congress of european society for medical oncology, esmo 2018'**:nc OR **'44th congress of european society for medical oncology, esmo 2019'**:nc OR **'44th esmo congress'**:nc OR **'60th annual meeting of the american society of hematology, ash 2018'**:nc OR **'61st ash annual meeting'**:nc OR **'american association for cancer research annual meeting 2017'**:nc OR **'american association for cancer research annual meeting 2019'**:nc OR **'american association for cancer research annual meeting, aacr 2020'**:nc OR **'american association for cancer research annual meeting, aacr 2022'**:nc OR **'annual meeting of the american society of clinical oncology, asco 2021'**:nc OR **'annual meeting of the american society of clinical oncology, asco 2022'**:nc OR **'annual meeting of the endocrine society, endo 2020'**:nc OR **'annual scientific meeting of the american college of gastroenterology, acg 2021'**:nc OR **'esmo congress 2021'**:nc OR **'esmo congress 2022'**:nc OR **'esmo immuno oncology congress 2019'**:nc OR **'esmo immuno-oncology congress 2022'**:nc OR **'esmo virtual congress 2020'**:nc OR **'iaslc 19th world conference on lung cancer'**:nc OR **'iaslc 2019 world conference on lung cancer (wclc)'**:nc OR **'jahrestagung der deutschen, osterreichischen und schweizerischen gesellschaften fur hamatologie und medizinische onkologie'**:nc) AND (**'conference abstract'**/it OR **'letter'**/it) | 152 |
| #7 | **nivolumab** AND **immune** AND **checkpoint** AND **inhibitors** | 14875 |
| #6 | #5 AND (**2006**:py OR **2008**:py OR **2009**:py OR **2010**:py OR **2011**:py OR **2012**:py OR **2013**:py OR **2014**:py OR **2015**:py OR **2016**:py OR **2017**:py OR **2018**:py OR **2019**:py OR **2020**:py OR **2021**:py OR **2022**:py) | 35546 |
| #5 | **nivolumab** | 47108 |
| #4 | #3 AND (**2014**:py OR **2015**:py OR **2016**:py OR **2017**:py OR **2018**:py OR **2019**:py OR **2020**:py OR **2021**:py OR **2022**:py) AND (**'case report'**/de OR **'case study'**/de) | 375 |
| #3 | #2 AND **'case report'**/de AND (**'conference abstract'**/it OR **'letter'**/it) | 478 |
| #2 | **ipilimumab** AND **immune** AND **checkpoint** AND **inhibitors** | 9259 |
| #1 | **'immune checkpoint inhibitors'**/exp OR **'immune checkpoint inhibitors'** OR ((**'immune'**/exp OR **immune**) AND (**'checkpoint'**/exp OR **checkpoint**) AND (**'inhibitors'**/exp OR **inhibitors**)) | 65158 |
