# Supplementary material for: Immune checkpoint inhibitors associated inflammatory disease of central nervous system: Case report and systemic review
Source: Medicine (Baltimore). 2025 Aug 1;104(31):e43552. doi: 10.1097/MD.0000000000043552 (PMC12324009; doi:10.1097/MD.0000000000043552)

**Figure S1.** Summary of Case Report;

Abbreviations: GMG = Generalized myasthenia gravis; IVIg = intravenous immunoglobulin; IVMP = intravenous methylprednisolone; ICIs = Immune Checkpoint Inhibitors.


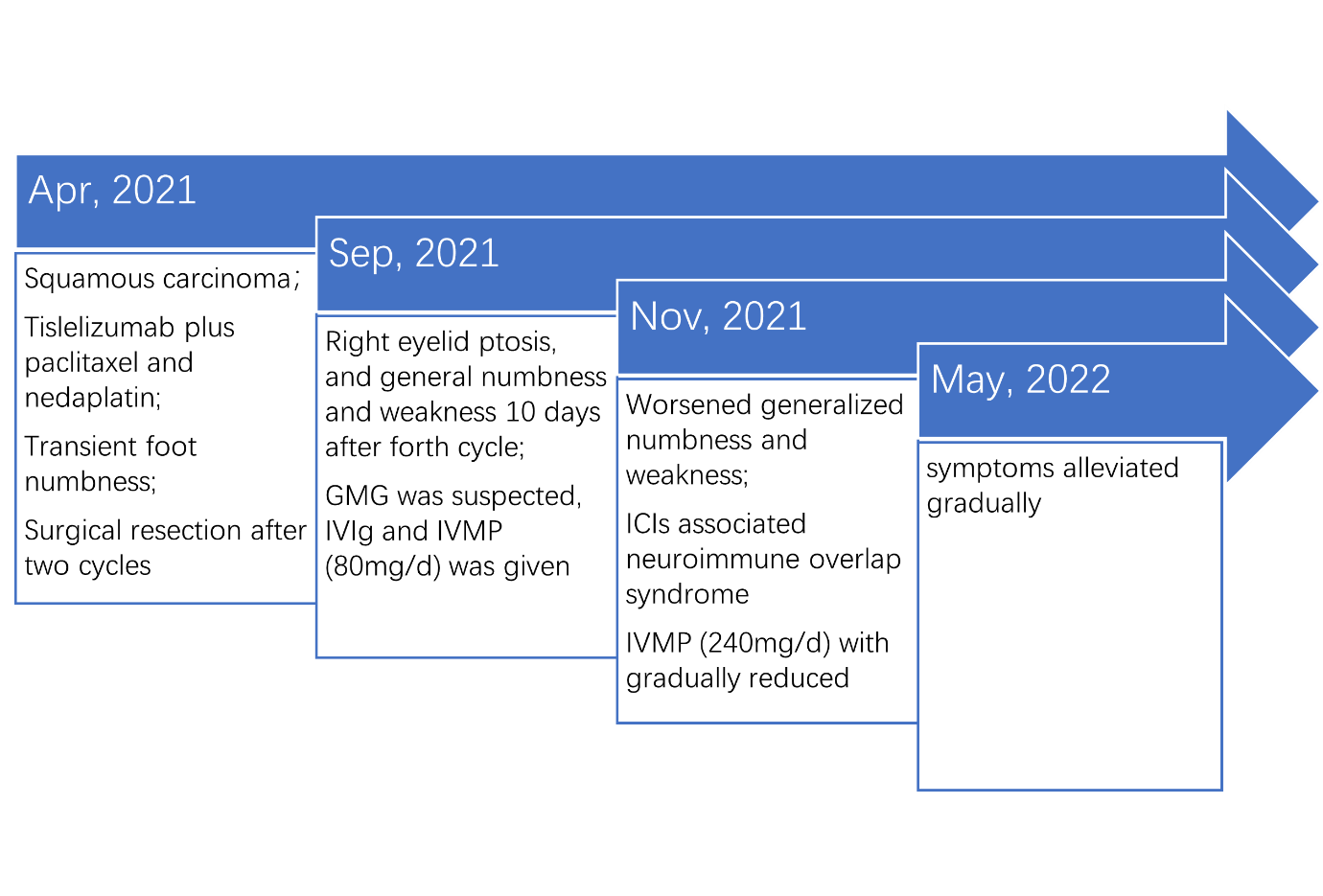

Supplement: Supplementary file 3 [file medi-104-e43552-s003.docx]
